# Adoption of Digital Health Technologies in the Practice of Behavioral Health: Qualitative Case Study of Glucose Monitoring Technology

**DOI:** 10.2196/18119

**Published:** 2021-02-03

**Authors:** Suepattra G May, Caroline Huber, Meaghan Roach, Jason Shafrin, Wade Aubry, Darius Lakdawalla, John M Kane, Felicia Forma

**Affiliations:** 1 PRECISIONheor Los Angeles, CA United States; 2 PRECISIONheor New York, NY United States; 3 Philip R Lee Institute for Health Policy Studies University of California San Francisco San Francisco, CA United States; 4 University of Southern California Los Angeles, CA United States; 5 School of Medicine Hofstra University Hempstead, NY United States; 6 Northwell Health New York, NY United States; 7 Otsuka Pharmaceutical Development & Commercialization Inc Princeton, NJ United States

**Keywords:** digital technology, chronic disease, blood glucose self-monitoring, diabetes self-management, real-time systems, mental illness, mobile phone

## Abstract

**Background:**

Evaluation of patients with serious mental illness (SMI) relies largely on patient or caregiver self-reported symptoms. New digital technologies are being developed to better quantify the longitudinal symptomology of patients with SMI and facilitate disease management. However, as these new technologies become more widely available, psychiatrists may be uncertain about how to integrate them into daily practice. To better understand how digital tools might be integrated into the treatment of patients with SMI, this study examines a case study of a successful technology adoption by physicians: endocrinologists’ adoption of digital glucometers.

**Objective:**

This study aims to understand the key facilitators of and barriers to clinician and patient adoption of digital glucose monitoring technologies to identify lessons that may be applicable across other chronic diseases, including SMIs.

**Methods:**

We conducted focus groups with practicing endocrinologists from 2 large metropolitan areas using a semistructured discussion guide designed to elicit perspectives of and experiences with technology adoption. The thematic analysis identified barriers to and facilitators of integrating digital glucometers into clinical practice. Participants also provided recommendations for integrating digital health technologies into clinical practice more broadly.

**Results:**

A total of 10 endocrinologists were enrolled: 60% (6/10) male; a mean of 18.4 years in practice (SD 5.6); and 80% (8/10) working in a group practice setting. Participants stated that digital glucometers represented a significant change in the treatment paradigm for diabetes care and facilitated more effective care delivery and patient engagement. Barriers to the adoption of digital glucometers included lack of coverage, provider reimbursement, and data management support, as well as patient heterogeneity. Participant recommendations to increase the use of digital health technologies included expanding reimbursement for clinician time, streamlining data management processes, and customizing the technologies to patient needs.

**Conclusions:**

Digital glucose monitoring technologies have facilitated more effective, individualized care delivery and have improved patient engagement and health outcomes. However, key challenges faced by the endocrinologists included lack of reimbursement for clinician time and nonstandardized data management across devices. Key recommendations that may be relevant for other diseases include improved data analytics to quickly and accurately synthesize data for patient care management, streamlined software, and standardized metrics.

## Introduction

### Background

In 2017, 46.6 million US adults—nearly 1 in 5—were living with mental illness, ranging in severity from mild to severe [1]. Nearly a quarter of these adults (11.2 million) were afflicted with serious mental illness (SMI), defined as a diagnosis of mental illness (eg, schizophrenia and schizoaffective disorders, bipolar disorder, or psychosis) that is persistent, disabling, and requiring specialized psychiatric treatment [1,2]. These patients have higher morbidity and mortality rates than individuals without SMI because of untreated and/or preventable chronic or infectious diseases [3,4]. In recent years, digital health technologies have been increasingly developed and recognized as care management tools for patients with SMI [5].

Digital health technologies and digital health interventions such as smartphone apps and wearable technologies can transmit data to health care providers. These technologies have enormous potential to improve care, offering patients, providers, and caregivers greater access to and information about illness management, treatment monitoring and medication adherence, and outcomes for a breadth of conditions, including SMI [5-7].

Yet, despite these potential benefits, the uptake of digital health technology in SMI has been low, and behavioral health practitioners have been hesitant to fully embrace digital health interventions for a variety of reasons. These include limited evidence and perceptions of efficacy, aversion to change, concern about added workflow, lack of appropriate reimbursement, and an increasingly overwhelming variety of features and sensors [8-10]. In the area of mental illness, there are added concerns, including stigma [11], privacy, and uncertainty regarding patient acceptance. In many respects, mental illness may derive the most benefit from new digital technologies as patient clinical evaluations are typically based largely on patient or caregiver self-report of symptoms [12]. In addition, the measurement of domains such as sleep, activity, social interactions, and medication ingestion in real time can be highly valuable to patients, clinicians, and caregivers [13].

Digital health technology has great potential to improve SMI patient outcomes and disease management. However, an understanding of the potential barriers and facilitators to adoption is warranted. How specific digital health technologies have been implemented and ultimately adopted (or not) in other therapeutic areas can serve as useful case studies from which lessons for future digital health implementation in SMI may be drawn. For example, a recent review of digital health technologies to manage hypertension found that the settings and context in which interventions are introduced as well as the individuals involved influenced adoption [14]. A 2017 study examining the implementation of mobile apps for patients to support postsurgical rehabilitation in orthopedics found that digital literacy and the impact of the intervention on outcomes and workflow need to be accounted for [15]. Another study examining digital health interventions to improve medication adherence in diabetes and hypertension found no conclusive evidence of improved adherence with technologies that incorporated features such as interactive voice response or telemonitoring [16].

The literature documents the introduction of digital health technology interventions across the treatment spectrum, especially how these may be used to manage chronic illness outside of the clinic [17]. However, most of these digital health interventions are still relatively new. To better understand the adoption of digital health technology interventions over a longer period whereby the technology has also evolved, we consider the case of how providers have collected data on patient glycemic marker hemoglobin A_1c_ (HbA_1c_) levels over time in diabetes care delivery. HbA_1c_ reflects the average blood glucose or blood sugar level in an individual over the past 3 months. In managing diabetes, physicians use HbA_1c_ to monitor how well a patient may be managing their diabetes. Historically, endocrinologists were reliant upon patient self-monitoring and patient or caregiver reports of glycemic events to inform diabetes management and treatment decision making. Over the past 20 years, digital health technologies, such as continuous glucose monitors (CGMs) and mobile glucose monitoring, support HbA_1c_ management by transmitting glucose levels to patients and clinicians and provide real-time insulin dose recommendations [18,19]. Each succession of new digital glucose monitoring technology has improved the quality, frequency, and relevance of monitoring glucose levels over time [20]. Such data have the potential to allow physicians to better understand a patient’s condition and assess treatment response in real time. In diabetes care delivery, endocrinologists play an important role in promoting adoption of and adherence to digital glucometer devices and are also key users of the technology, as the devices provide data to be used in clinical decision making.

### Objectives

This study sought to investigate the trajectory of technology adoption in the monitoring and treatment of another chronic disease—diabetes—to identify issues and facilitate improved adoption that may be relevant to the adoption of digital health interventions within the field of behavioral health. By adopting a qualitative case study approach, this study aims to understand the key facilitators to and barriers of clinician and patient adoption of digital glucose monitoring technologies for diabetes and identify lessons learned that could be relevant for the treatment and monitoring of other chronic conditions, including SMI.

## Methods

### Study Design

Using qualitative data collection methods, we conducted focus groups with endocrinologists in 2 major metropolitan regions of the United States. Through focus group–guided discussions with these clinicians, we elicited perspectives of, and reports of experiences with, the implementation of digital glucose monitoring technologies in endocrinology practices over the past 2 decades and how such technologies impacted day-to-day clinical practice.

### Participant Recruitment

We engaged a medical market research firm to identify and recruit potential participants from their panel of specialty health care providers. Bias was minimized by using probability sampling to select potential clinicians to receive a study invitation in 2 major metropolitan regions of interest in the United States. A convenience sample of endocrinologists treating patients with diabetes was invited by email to participate in the study. Physicians were eligible to participate if they (1) were board certified in endocrinology and currently practicing medicine, (2) had spent at least 80% of full-time equivalent in clinical practice treating patients with diabetes, and (3) had reported a minimum of 10% of their patient panel used a digital blood glucose monitoring device. We excluded physicians who had not been in clinical practice for a minimum of 10 years to ensure that participants could provide a relevant historical perspective on the evolution of digital glucose monitoring and its implementation in clinical practice.

Endocrinologists who met the basic study eligibility requirements were contacted by telephone and screened with a study-specific questionnaire to confirm eligibility and capture demographic data. Participants were compensated for study completion.

The Advarra Institutional Review Board (IRB) reviewed the study protocol, informed consent documents, and focus group discussion guides. The Advarra IRB determined that the study met the requirements for exemption from IRB oversight and granted a waiver of exemption from review.

### Data Collection

Two 90-min focus groups were conducted in December 2018 in 2 large metropolitan regions in the United States. One member of the study team with doctoral training in qualitative research methods (SM) conducted the focus groups using a semistructured focus group discussion guide. A literature review was performed to inform the development of the discussion guide. Sample discussion guide questions are detailed below (Textbox 1). Focus groups were audio-recorded and transcribed verbatim. In addition to the focus group discussion, 2 members of the study team (CH and MR) recorded observational field notes.

**Sample discussion guide questions.**

Perceptions of and experiences with diabetes management before digital glucose monitoring technologies

- Before the introduction of continuous glucose monitors (CGMs), what was the most optimal way to manage patients with diabetes, that is, how did you establish baseline glucose profiles, monitor glucose levels, etc?
- How did you first introduce self-monitoring or home glucometers to your patients?
- How was the transition from patient self-monitoring to continuous glucometers or technology?

Glucose monitoring technology adoption

- What prompted discussions on the need to adopt continuous glucose monitoring devices in your respective practices?
- What benefits did you anticipate and why?
- What potential disadvantages did you anticipate and why?
- How do you introduce the idea of continuous glucose monitoring technologies to patients?

Barriers of and facilitators to adoption and implementation

- What considerations, processes, or key personnel facilitated the decision to adopt glucose monitoring devices in your respective practices?
- How much personnel time or other resources have you or your practice invested into education and training?
- How much time do you or your staff devote to reviewing and/or discussing data from glucose monitoring technologies with patients?
- How do data from glucose monitoring technologies affect your clinical decision making?

Recommendations for strategies and approaches to facilitate adoption and uptake

- Looking back at the implementation, what do you think have been the most effective strategies and approaches for glucose monitoring technology adoption and uptake in your clinical practice?
- What challenges do you see to future adoption of glucose monitoring technologies in practice?
- What recommendations or advice do you have to increase glucose monitoring technology adoption?

### Data Analysis

The constant comparative method was used to analyze the transcripts, facilitated by using MAXQDA qualitative data management software program (MAXQDA12; VERBI, GmbH) [21]. Our analytic approach used a comprehensive review of the transcripts incorporating the constant comparative method. Data analysis was consistent with the principles of thematic analysis outlined by Glaser and Strauss [21]. This approach to analysis involves identifying similarities and differences in the ways participants discussed their perspectives of and experiences with digital glucose monitoring technology adoption and is widely used in qualitative research [22]. The analysis involved 4 stages: (1) initial coding was undertaken by members of the project team with graduate training in qualitative data analysis (CH, SM, and MR). Each team member individually reviewed the data to develop a general overall impression of the data content, outline preliminary descriptive categories, and develop a preliminary coding framework. (2) Codes generated during preliminary coding were further refined and defined into descriptive coding categories, resulting in a final coding framework with a set of codes generated inductively and derived from the original interview questions. (3) The codes were applied to each focus group transcript. To assess the degree of coding consistency during this phase, one member of the project team (SM) reviewed all coding to ensure that the coders understood and applied the codes in accordance with the definitions in the coding dictionary. (4) Using the constant comparative method, concepts, themes, and patterns emerged from the data. These were integrated and refined into meaningful groupings by the team and based on consensus. The analysis provided insight into participants’ experiences, motivations, and actions regarding treatment decision making related to glucose monitoring technology, yielding the salient themes presented in the results.

## Results

### Participant Sample

A total of 10 endocrinologists participated in the study. Most (6/10, 60%) of the sample was male, and the mean age was 48.6 (SD 5.9) years, with an average of 18.4 (SD 5.6) years since receiving their medical license. Of note, most of the sample (8/10, 80%) reported that more than half of their diabetic patient population used digital glucose monitoring technologies. Participant demographic characteristics are detailed below (Table 1).

**Table 1:** Participant demographics (N=10).

Variable			Participants, n (%)
Age (years), mean (SD)			48.6 (5.9)
**Gender, n (%)**			
	Male	6 (60)	
	Female	4 (40)	
**Race, n (%)**			
	African American	0 (0)	
	Asian	5 (50)	
	Mixed or Other	0 (0)	
	Native Hawaiian or Pacific Islander	1 (10)	
	White	2 (20)	
	Missing or refused	2 (20)	
**Hispanic ethnicity, n (%)**			
	Hispanic	1 (10)	
	Not Hispanic	9 (90)	
	Missing or refused	0 (0)	
Board certification in endocrinology, n (%)			10 (100)
Years since graduated medical school, mean (SD)			21.2 (5.8)
Years since obtaining medical license, mean (SD)			18.4 (5.6)
**Primary practice setting, n (%)**			
	Academic medical center	1 (10)	
	Endocrinology group practice	1 (10)	
	Group model health maintenance organization	3 (30)	
	Multispecialty group practice	3 (30)	
	Solo practice	2 (20)	
**Years prescribing blood glucose monitors, n (%)**			
	2-5	1 (10)	
	6-10	5 (50)	
	More than 10	4 (40)	
**Proportion of patients with diabetes using a digital glucometer (%), n (%)**			
	Less than 10	0 (0)	
	10-25	2 (20)	
	25-50	0 (0)	
	50-75	2 (20)	
	More than 75	6 (60)	

### Key Findings

#### Digital Monitoring Technologies Changed the Treatment Paradigm in Diabetes Care Delivery

Before the availability of digital glucose monitoring technologies, clinical management of diabetes was predominantly reactive management by endocrinologists and largely based on laboratory test results, clinical signs, and patient self-reporting. Glucose testing required a waiting period, whether for urine strips (60 seconds) or blood finger pricks (a few seconds), which served as a barrier to patients regularly checking their levels [23].

Participants in this study noted that patient demand, clinical recommendations, and research, such as those presented at national professional conferences, drove the transition from laboratory tests and urine strips to digital glucose monitoring technologies. In addition, adoption was heavily influenced by Food and Drug Administration approval of digital glucose monitoring devices and direct-to-consumer advertising; the former was important for validating physician trust in the technology, and the latter was influential in generating patient demand for digital glucose monitoring technologies.

Despite the demand, participants initially were apprehensive about the reliability (ie, accuracy of data) of glucose monitoring technologies. As broad adoption occurred and the devices became easier to use and more reliable, skepticism was overcome as described by one endocrinologist:

I mean, you're always skeptical when something new comes out. When the first pumps came out...people then didn't want to wear it, after wearing it for a little bit. And then it was uncomfortable. And so, if the person can't tolerate wearing something, it's just not going to work... That really dissuades people from wanting to start it, for one thing. So that was a concern. But as people get more tech savvy, it's less and less difficult. I mean, it's taken me a while but in the beginning, it was really hard. So it's definitely been an evolution.

#### Digital Monitoring Has Improved Patient Outcomes and Facilitated More Effective Care Delivery and Patient Engagement

Before the introduction of digital glucose monitoring, efforts to reduce the risk of hypoglycemia were challenging for both endocrinologists and patients. Endocrinologists only had access to glimpses of a patient’s glucose level at specific points of time, as opposed to a more complete picture of HbA_1c_ levels that are provided by continuous glucose monitoring technologies, especially information on whether blood sugar is trending down or trending up [20].

Participants in this study described the ways in which digital glucose monitoring technologies have provided a range of benefits for their patients. A primary benefit was the potential for the digital glucose monitoring devices to raise provider and patient awareness of glycemic events, thereby reducing the risk of disease-related morbidity and mortality.

Historically, endocrinologists depended on patient self-reporting and laboratory testing to inform diabetes management and treatment decision making. Today, diabetes technologies, such as continuous glucose monitoring and mobile glucose monitoring, can support HbA_1c_ management by transmitting glucose levels to patients and clinicians and provide real-time insulin dose recommendations [10]. Such technologies quantify glucose levels over time and provide insight into patterns and trends that allow the clinician to better understand a patient’s diabetes and gauge how the patient is responding to treatment:

...in the last decade or so, a revolution happens in endocrinology...before then, we are just doing nothing because we have just a few insulin [options] ...And we're maybe lucky to get A1C of eight and you are happy by that. And patient was frustrated, doctor was frustrated because we cannot do much more. Just that's it. We have very much more advanced insulin and several type of class of medication now. And at the same time, we have very advanced technology. It's completely a game-changer now. Easily we can get A1C of seven or less, easily. And that's why doctors are more happy, patients are more happy.

As endocrinologists are able to see accurate data in real time, they reported not having to rely as much on subjective patient or caregiver reports to aid in clinical decision making. The availability of continuous data also allowed providers to identify patterns or trends around glycemic events in their patients:

But truly, it's just more information. Prior to that...when we used NPH [neutral protamine Hagedorn insulin], everybody was getting low and we just never checked them. And they didn't check themselves. I feel like it's changed. It's going to change longevity, I mean, in terms of their lifespan. And it's going to make a huge difference. So I feel like if that's the standard of care now, just people will just jump on board.

Participants reported that glucose monitoring technologies can also ease caregiver burden by allowing caregivers to monitor patients’ glucose levels, which is especially important for patients susceptible to hypoglycemic events:

Now the technology…you can assign a surrogate. They could be assigned and they could be informed about blood sugars before even the patient recognizes. If you have a continuous glucose monitoring and you have those uploaders and you assign a couple of people, maybe a teacher or a parent. He's at work and he can alert the teacher. I see [inaudible] going down. You can do it remotely. So that's fabulous for usually type 1 diabetic patients.

With the widespread adoption of digital glucose technologies, providers reported being able to customize and calibrate treatment—and more aggressively if need be—thereby improving clinician decision making and patient care delivery.

#### Digital Monitoring Technologies Have Introduced New Challenges for Clinical Care Delivery

The practice environment and associated availability of resources emerged as a key determinant of digital glucose monitoring technology adoption and use in clinical practice. Participants discussed that while the availability of data streamlined certain areas of diabetic patient management, these devices often generated more work and responsibilities than anticipated, such as data management and obtaining coverage approval and reimbursement.

Participants emphasized that both they and their patients experienced a significant burden and bureaucracy associated with insurance and reimbursement coverage of the devices and associated visits. Payer coverage of digital glucose monitoring technologies is highly varied, and most payers require preapproval for the device and all sensors, which may only be applicable for a limited duration (days or weeks at a time), requiring endocrinologists to repeatedly complete the burdensome process of obtaining preapproval.

Participants noted that there are very few billing codes for these types of digital health monitoring devices; therefore, providers may not receive compensation for the time they devote to educating patients and addressing general device-related management issues. In the following narrative, a participant describes the time he must spend obtaining insurance approval for broken equipment while alluding to the fact that he is not being reimbursed for the time he spends:

And then just the time because it's a durable medical piece of equipment, the time. Things break, the clip, the tube gets twisted...Just dealing with all of that takes a lot more time than before we had it. So I mean, patients will always be – they're short one tube being sent but the insurance is not going to allow them to have it. So I mean, then you're on the phone trying to make an exception.

A related challenge is the lack of dedicated staff at some practices to support patients with digital glucose monitoring devices. Participants noted the inherent limitations of the devices, including technology failures, device repairs, and patient education and troubleshooting. In the absence of staff who can provide support with these devices, providers are left to deal with these issues directly, taking unplanned time from their busy schedules:

It's so important if you're lucky enough to have CDE [Certified Diabetes Educator] or a group of people to help you. That their only focus is the technology. So if you've got someone like that on your side, it makes a huge difference in terms of who's going to get started and who's going to get used to their equipment quicker and who's going to feel comfortable.

Although the data generated by digital glucose monitoring technologies have improved the health of their patients, participants noted that the data generated are often not easily actionable for 2 reasons. First, the sheer amount of data the devices generate cause *data overload* for the providers. Second, the data are presented in a form that offers limited context about the patient and their diabetes management. These 2 issues make it challenging for providers to analyze and synthesize data during a short patient visit:

It takes 20 minutes just to download and the next patient is there. Two other patient already showed up. And this is time-consuming. Just adjusting the pump, doing the download.

Participants noted an overall lack of funding and resources to support all of the structural challenges described: coverage and reimbursement, data management, and patient support.

#### Case Study Lessons: Recommendations and Opportunities for Increasing Uptake of Digital Health Technologies

In the focus group discussions, participants were explicitly asked what recommendations they would make to increase uptake of digital health technologies more generally, based on their experiences with digital glucose monitoring technologies. Participants in this study felt that if digital health technologies were going to have a meaningful and effective role in diabetes management, tangible steps needed to be taken by providers and payers to bridge gaps in care and reimbursement policy, which in turn would increase the access to and uptake of the technologies. These recommendations are not limited to the field of endocrinology and are applicable to the incorporation of digital health technologies in other therapeutic areas as well. These included the following recommendations:

- Expand coverage and streamline approval processes: Participants recommended expanding coverage for patients by following review and approval processes in place for specialty prescription drugs. Specifically, the endocrinologists believed that insurance review and coverage approvals for high-cost specialty drugs were far more streamlined than the review processes that payers require for interventions that fall under a device classification. From their perspective and experience, device approvals appear to invite more scrutiny by insurers than specialty drug approvals.
- Provide reimbursement for clinician time: Participants noted the substantial amount of time they or their staff spent supporting patients on glucose monitoring technology use and device maintenance. In addition, although endocrinologists appreciated the data that digital health technologies generated, they were not able to synthesize the vast amount of data downloaded during each office visit, which is often the only time they could bill for reviewing the data. To address both challenges, they recommended reimbursement for and appropriate billing codes to document the time devoted to device support and data review.
- Streamline software and data management across device platforms: Participants reported that a lack of standardization across digital glucose monitoring devices and corresponding platforms has proven burdensome for clinic staff and clinic operations. Specifically, endocrinologists indicated that standardized metrics and a streamlined transfer of digital monitoring technology data to their medical records would make clinic visits more efficient and provide the clinician more time to review data with the patient during the office visit. Several providers in this study indicated that it would be most beneficial to have the data from digital glucose monitoring technologies integrated with their electronic medical record software. In addition, in the absence of appropriate reimbursement noted above, they recommended that manufacturers develop algorithms to assist in analyzing and displaying clinically relevant and actionable information for physicians during the clinic visit.
- Digital health technologies should be customized to patient needs: Participants explicitly noted that digital health technologies should not be a one-size-fits-all approach to care management. Specifically, it is critical to assess patient receptivity to technology and tailor the digital health technology recommendation accordingly. Participants explained that some technologies are more advanced than others, such as those that synchronize data to a mobile phone app or that, in the case of glucose monitors, provide alarms if the patient’s glucose levels are outside a given range. Participants emphasized that some patients prefer more complex technologies, whereas others are better suited to simpler devices. Patient reluctance to try new or novel digital technology can often be countered by offering patients free trials, allowing the patient an opportunity to experience the technology without immediately committing to use.

## Discussion

### Principal Findings

Digital health technologies provide an opportunity to promote better treatment decision making and measure and monitor patient outcomes in real time. The objective of this study was to review the adoption of digital glucose monitoring as a case example of digital health penetration to identify lessons learned that may be applied to the adoption of digital health technologies in the realm of behavioral health. The endocrinologists participating in our study described the spectrum of benefits and challenges they experienced and detailed specific recommendations for how digital health monitoring technologies may be applied to the clinical management of other chronic conditions, including behavioral health. From their reported perspectives, digital health monitoring has improved care delivery by providing data about patients that can help to better monitor patient adherence and response to therapy and personalize treatment in a manner that best optimizes patient outcomes.

At the same time, participants in this study expressed concerns over the sheer volume of data that digital health technologies have generated and the urgent need to find solutions that can help to relieve physician burden and not impede the care they deliver. Such solutions include removing barriers to insurance approval, expanding categories of reimbursement, and developing software tools or algorithms that can help providers and patients navigate and understand data. Participants recommended synthesizing data into brief, tailored reports that can be used to inform clinical management decisions during an office visit. Separately, payers do not yet provide sufficient reimbursement for the provider time required to prescribe and manage digital technologies, which limits the opportunities for full integration of these devices into clinical care. Being able to quantify the long-term clinical and economic benefits of digital health technologies may be one mechanism by which reimbursement can be attained. Despite the challenges encountered, the endocrinologists in this study described how the continuous improvements in technology and the clear improvements in clinical outcomes for patients that they had observed over the years led to widespread adoption of the devices in clinical practice.

### Limitations

Although this study has yielded suggestive findings, it is not without sampling-related limitations that merit consideration before interpreting the study and its implications. The findings represent the views of a small convenience sample of individuals who may be more vocal than the general endocrinologist population. However, similar themes were revealed and described in both focus groups, suggesting that the patterning of data may be similar in groups with similar characteristics. It should be noted that the study participants were self-selected in that they volunteered for the study. In addition, this study was limited to 10 endocrinologists practicing in 2 large metropolitan regions and may not represent experiences and perceptions of other types of providers who serve patients with diabetes (eg, primary care physicians or nurse practitioners) or providers who practice in different types of socioeconomic settings or geographic regions. Larger-scale work is needed to establish the generalizability of the findings.

Despite these limitations, this study provides preliminary qualitative data from a group of highly experienced clinicians on the key factors associated with successful implementation of digital monitoring technologies and strategies to mitigate and overcome potential barriers to uptake.

### Comparison With Previous Studies

Previous studies have assessed barriers to and facilitators of the adoption of glucose monitoring technologies from the perspective of patients, their caregivers, and providers. Studies with patients and caregivers have found that patients who used glucose monitoring technologies perceived better control of their diabetes and improved confidence in controlling glucose levels, and patients were more likely to use glucose monitoring technologies if recommended by their clinician or family members [24,25]. Patient-reported barriers to use include high cost and limited reimbursement, device discomfort, disruptive alarms, unfamiliarity or distrust with the technology, and added burden to calibrate the device and understand and respond to data [24-29]. Several studies have also explored provider-level barriers and facilitators associated with the adoption of glucose monitoring technologies. In these studies, providers reported having insufficient time to interpret data, inadequate reimbursement or bureaucratic reimbursement processes, perceived issues with technology accuracy and security, and a lack of ancillary resources as barriers to adoption [30-32]. Although these studies provide a basis for understanding provider challenges and other barriers, surveys were employed to collect data, and the studies were not designed to explore, in depth, any nuances related to the adoption of technologies.

Before this study, specific gaps in the literature included (1) how providers learned about new technologies and the decision to adopt into clinical practice, (2) how providers decided to recommend technologies to their patients, and (3) how providers approached patient resistance to new digital health technologies. Although our study findings generally align with those of previous published studies, the specific processes by which glucose monitoring technologies have actually been adopted into clinical practice have been more fully elucidated in this study, including the recommendations that providers have for increasing future uptake of digital health technologies. This study also aligns with findings by Fonseca et al [33] who concluded that the adoption of CGM could be increased through standardized and tailored reporting coupled with expanded reimbursement to cover both the cost of the device (to patients) and the time spent advising patients on using the device (for providers). A recent report from the American Medical Association underscores this study’s findings on digital health technology adoption by physicians. Specifically, doctors are reported to assess the feasibility of using new digital tools based on 3 factors: effort (how seamless the integration of the innovation be), outcome (value to patients), and finance (how much it will benefit practice). The report also emphasized that any new tools or solutions must also address the issues of coding and coverage [34].

### Lessons Learned and Implications for Future Research

Future research should consider the benefits of and challenges to the adoption of digital health technologies in other therapeutic areas, such as behavioral health. However, for meaningful and effective uptake to occur, tangible steps must be taken to substantively apply lessons learned. On the basis of our findings, some lessons learned that may help to bridge the gaps in care and increase adoption and adaptation of digital health technologies in the practice of behavioral health include:

- Generate robust evidence on how digital health in SMI affects physician, patient, and caregiver decision making: for instance, a large share of mobile health apps is dedicated to treating SMI [35]. For many of these apps, evidence demonstrating treatment efficacy and use for physicians is limited [36]. Some digital health technologies in the SMI space, however, have shown the potential to improve physician decision making and decrease health care costs [37,38]. In fact, new technologies include both pharmaceutical treatment and digital sensors [39].
- Expand coverage and streamline approval processes: our participants described a movement from reactive management and reliance on infrequent self-reporting to more timely and accurate assessment through the use of digital technologies. They also noted the value of improved provider and patient awareness and communication and the opportunity to reduce caregiver anxiety and burden, all of which can ultimately improve patient outcomes. However, the amount of time practitioners must devote to navigating administrative challenges for patients’ digital monitoring technologies can impede uptake. If new technologies are shown to be cost-effective, access to digital technologies will be worth the additional cost.
- Provide reimbursement for clinician time and resources for staff training and patient support: providers in this study who had dedicated resources and personnel to support digital monitoring technologies in their practices observed positive impacts and higher long-term adherence and use. This included clinician review and synthesis of device data, providing support for securing coverage approvals, patient education and training, and continued device monitoring, maintenance, and patient follow-up. However, not all providers had access to dedicated personnel and resources. Reimbursement for clinician time and associated resources could facilitate the expanded adoption of digital health technologies. The cost of clinician time and staff training should be incorporated into any economic model of new treatment value.
- Customize digital health technology tools to the patient: recognizing that digital technologies are not a one-size-fits-all solution, it is critical that the technology fits each individual patient’s needs and that incentives for providers are aligned with the heterogeneity of patients’ particular preferences and needs, rather than clinic- or physician-level metrics.

Our findings suggest that there are substantial challenges to effectively incorporating digital technologies within the context of clinical care; however, these challenges are not insurmountable if identified and addressed up front. All of these considerations play a critical role in potentially improving the management of patients with SMI, where infrequent and subjective assessment, recall bias, problems with medication adherence, and enormous caregiver burden are significant unmet needs. Additional focus groups with other physician specialties will also be useful as they face similar decisions regarding if, when, and how to adopt new digital health technologies into real-world treatment of patients with SMI.

### Conclusions

Endocrinologists reported that digital glucose monitoring technologies facilitated more effective, individualized care delivery and improved patient engagement and health outcomes in diabetes care delivery. However, challenges faced by clinicians included a lack of reimbursement for clinician time and nonstandardized data management across devices. Key recommendations that may be relevant for other diseases include improved data analytics to quickly and accurately synthesize data for patient care management, streamlined software, and standardized metrics. Future research should examine the extent to which these learnings are relevant to the adoption of digital health technologies for other therapeutic areas. Further research is also warranted to systematically quantify the challenges described in this study in a larger sample of clinicians. The results suggest strategies and areas of focus for stakeholders to mitigate barriers and bridge gaps in digital monitoring technology uptake.

## Abbreviations

CGM: continuous glucose monitor
HbA_1c_: hemoglobin A_1c_
IRB: Institutional Review Board
SMI: serious mental illness
